# Editorial: Green and sustainable chemistry editor’s pick 2024

**DOI:** 10.3389/fchem.2024.1546377

**Published:** 2025-01-07

**Authors:** James Clark

**Affiliations:** Green Chemistry Centre of Excellence, University of York, York, United Kingdom

**Keywords:** waste feedstocks, waste water, biomass, calcium carbonate, clean synthesis

Green and Sustainable chemistry is at the heart of the global movements towards a circular economy and a more sustainable society. Green Chemistry became a recognised term 30 years ago and largely built on many years of process chemistry improvements that led to safer and more efficient chemical manufacturing. The early movement was strongly focussed on avoiding where possible, the more hazardous chemical substances and reducing toxic chemical waste. While these remain as core components, there is now a more holistic view of the chemical space with feedstocks, process and products sharing the limelight. Frontiers Green and Sustainable Chemistry seeks to embrace all key stages in the life-cycle of chemical products and including key subjects including renewable resources and the valorisation of waste, clean synthesis and alternative technologies, and product performance, as well as environmental impact and recyclability. We seek high quality research articles that address these subjects in the context of important products across all key sectors, including advanced materials, pharmaceuticals and other bio-actives, electronics, and home and personal care products. We have selected 10 outstanding research articles from 2024 that reflect these principles and values.

In the article *Transformation of struvite from wastewater to a hydrogen fuel storage compound ammonia borane*, the interesting and important future source of hydrogen, ammonia-borane is synthesised from wastewater (Dingra et al.). This not only opens the door to a new use of a widespread waste, it can also help alleviate the growing concerns about adequate treatment of wastewater.

A more widely recognised use of organic wastes is their pyrolysis to produce useful oils, such as those that can be co-fed with traditional crude oil feedstocks. However, the biggest problem with such pyrolysis oils is often their poor quality. In *Processing renewable and waste-based feedstocks with fluid catalytic cracking: Impact on catalytic performance and considerations for improved catalyst design*, the optimisation of solid catalysts in FCC is shown to enable pyrolysis oils to be used as replacements for fossil oils with little impact on performance (Mastry et al.).

One of the largest volume but little exploited natural resources is lignin which remains more of a waste than a feedstock despite its chemical potential. In *Biocatalytic selective acylation of technical lignins: a new route for the design of new biobased additives for industrial formulations*, technical soda lignins are chemically modified using a biocatalytic process (Sarieddine et al.). The resulting materials have useful properties that suggest possible real industrial application value.

Many modern vaccines require the addition of adjuvants which are chemical entities that can induce a strong but controlled immune response. Despite their rapidly growing value, the rate of innovation of vaccine adjuvants has been very slow. In *Cleaner synthesis of preclinically validated vaccine adjuvants*, synthetic glycophospholipids have been developed (Romerio and Peri). These not only have promising activity and low toxicity, they can be made using good clean synthesis techniques including regio- and chemo-selective reactions.

The valorisation of biomass feedstocks is likely to become a vital part of the future chemical industry and we need to prove alternative technologies for this that go beyond traditional methods like pyrolysis. In the review article, *Mechanochemistry and oleochemistry: a green combination for the production of high-value small chemicals*, the little utilised mechanochemistry technique for oleochemistry is demonstrated in a number of recent articles (Len et al.). This includes original work aimed at creating new and efficient routes to important products including biodiesel, benzoxazine and solketal.

Deep eutectic solvents are attracting increasing interest as green solvents but their use in biocatalysis has been little reported. In *Harnessing the potential of deep eutectic solvents in biocatalysis: design strategies using CO*_*2*_ *to formate reduction as a case study*, these fascinating solvents are successfully demonstrated in the important reduction of carbon dioxide to formate (Logarušić et al.). This is achieved by a powerful combination of experimental screening and computational tools.

The removal of lead from water is an important clean water strategy. In *Improvement of magnetite adsorption performance for Pb (II) by introducing defects*, the ability of natural magnetite to adsorb lead is improved by surface defect engineering specifically by calcination under argon (Li et al.). The creation and effects of surface vacancies are studied by equilibrium and kinetic adsorption experiments.

Organic pollutants are of increasing concern to water purity and are becoming more common with the growth of many industries. Among the most worrying are organic dyes which are currently dumped in multi-thousand ton quantities around the world. In *Kinetics and thermodynamics investigations of efficient and eco-friendly removal of alizarin red S from water via acid-activated Dalbergia sissoo leaf powder and its magnetic iron oxide nanocomposite*, a new cost-effective natural adsorbent is proven for the adsorption of the hazardous dye alizarin red S from water (Nawaz et al.).

The complex behaviour of pollutants in water needs good mathematical modelling to aid field remediation planning. In *Modelling and optimization study on degradation of organic contaminants using nZVI activated persulfate based on response surface methodology and artificial neural network: a case study of benzene as the model pollutant*, the degradation effects of activated persulfate on the common groundwater contaminant benzene are modelled and then used to optimise the process parameters and maximise benzene removal (Luo et al.).

Calcium carbonate is one of the most common natural substances on our planet and is made even more important because of its role in helping to abate the CO_2_ crisis and help combat ocean acidification. We need to make more use of this compound. In *Improvements in the utilization of calcium carbonate in promoting sustainability and environmental health*, both well-known and less well-known applications for CaCO_3_ are described (Comes et al.). Utilization of pre-consumer and post-consumer recycled calcium carbonate is on rise and can help customers achieve their circular economy objectives.

